# Magnitude and Predictors of Health Care Workers Depression During the COVID-19 Pandemic: Health Facility-Based Study in Eastern Ethiopia

**DOI:** 10.3389/fpsyt.2021.654430

**Published:** 2021-07-15

**Authors:** Tesfaye Assebe Yadeta, Yadeta Dessie, Bikila Balis

**Affiliations:** ^1^School of Nursing and Midwifery, College of Health and Medical Sciences, Haramaya University, Harar, Ethiopia; ^2^School of Public Health, College of Health and Medical Sciences, Haramaya University, Harar, Ethiopia

**Keywords:** health worker, depression, COVID-19, eastern, Ethiopia, pandemic

## Abstract

**Background:** Depression of health care workers was related to work absences, resignations, and poor work performance, affecting the quality of patient care and the health care system. The Coronavirus disease pandemic has had an effect on the mental health of health care workers. Health care workers are facing challenges that can be stressful, overwhelming, and cause strong emotions, may put them at higher risk to develop depression. There is limited evidence that assesses health care workers' depression and its associated factors in the study area during the Coronavirus disease pandemic. Therefore this study aimed to assess depression and associated factors among health care workers in eastern Ethiopia.

**Method:** The cross-sectional study design was conducted from October 26th to November 15, 2020. A total of 265 health care workers from 10 health facilities participated. Patient Health Questionnaire was used for the collection of depressive symptoms. The data were analyzed by using STATA version 14 software. To assess the association between depression and the predictors Adjusted Odds Ratio along with a 95% confidence interval was estimated by using logistic regression analysis. A statistical significance was declared at *p*-value ≤ 0.05.

**Results:** Of the total 265 study participant, 176 (66.4%) and 95% CI: 60.4%, 71.8% of them reported depressive symptoms. Of 176 reported symptoms of depression 27.9, 24.1, 9.4, 3.7, and 1.1% were had minimal, mild, moderate, moderate-severe, and severe depressive symptoms respectively. The multivariable logistic regression analysis revealed the odds of depression were 2.34 times higher among female participants compared to male participants (AOR: 2.34, 95%CI: 1.09-5.02). In addition, the odds of depression for participants who perceived susceptibility to COVID-19 was 4.05 times higher among their counterpart (AOR: 4.05, 95%CI: 1.12-14.53).

**Conclusions:** Health care workers who experienced depression in the study was high. Health care workers' mental health needs to be protected during the COVID-19 pandemic. Female health care workers and health care workers perceived susceptibility of COVID-19 need attention.

## Introduction

Since the World Health Organization (WHO) declared coronavirus disease 2019 (COVID-19) a pandemic, it became a major challenging public health problem worldwide (1). Globally, many countries have been affected by the pandemic, as of October 27, 2020; there were 43,873,412 recorded cases and 1,166,039 fatalities (1). During the same period, 93,707 cases and 1,437 fatalities were recorded in Ethiopia (2). Similarly, more than 10,000 health care workers (HCWs) in the 40 countries of Africa have been infected with corona viruses disease (COVID-19) (3).

With the ever-growing number of confirmed and suspected cases, the workload of health care workers (HCWs) has been overwhelming (4). The long and irregular hours of such continuous and heavy volumes of work have the potential to trigger depression among HCWs (5–8). Safety measures like lockdown and physical distancing recommended by the world health organizations to limit the spread of pandemics among the public, but health care workers (HCWs) are left exposed (9). Globally, the prevalence of depression among HCWs range from 18 to 72% (10–16). Also, they are burdened with emotionally challenging interactions with the sick and potentially dying persons, fearing for their and families' health, and subject to occupational overload due to staff shortages and -insufficient personal protective equipment (17).

Many studies documented as HCWs face physical exhaustion, sleep disruption, fear, emotional disturbances, feeling vulnerable, loss of control, changes in working patterns/routine, feelings of personal danger, being isolated, lacking necessary supplies to conduct their work due to the COVID-19 pandemic (4, 18–20). This may increase their risk of depression, suicide, and burnout. Depression symptoms were related to work absences resignations and poor work performance, affecting patient care and the healthcare system (15, 21).

The studies identified feeling susceptible to contracting COVID-19, family/peers encouraged face mask use, access to adequate supplies of personal protective equipment (22), females sex, severe stress (23), having a chronic disease, and suspected or confirmed with COVID-19 infection were associated with depression among health care workers during COVID-19 pandemic (24). As a result of the ongoing COVID-19 pandemic, health care workers are fronting enormous worry due to the heavy load of cases. In many cases, they work increasingly long hours, often with limited resources and uncertain infrastructure. Thus, it is important to check on the mental health of caregivers (16).

Ethiopia reported the highest number of COVID-19 confirmed cases in East Africa. The number of confirmed COVID-19 confirmed cases and new deaths are alarmingly increasing. The number of the case at the end of December was 124,265 while on April 13 it was a surge to 232, 512 which was almost double with 4 months. The number of death at the end of December was 1,923 and almost double on April 13, 2021, 3,230. Eastern part of Ethiopia is a peripheral area sharing international borders were a greater number of COVID-19-related deaths. This may overwhelming and predispose the HCW to depression (25).

The study suggests that HCWs with depression benefit from care involving medical and psychological interventions (26). World Health Organization identified protecting the mental well-being of healthcare workers caring for people with COVID-19 has been identified as crucial for the long-term capacity of the HCWs (27). There is a limited study in Ethiopia that assesses the magnitude of depression and associated factors among health care workers during COVID-19. In addition, studies revealed HCWs experiencing depression were not planning to seek help and 30% were not aware of workplace help programs. This may be due to a lack of information on the prevalence and associated factors of depression. Therefore, this study aimed to assess the magnitude of depression status and associated factors among health care workers during the COVID-19 pandemic in the Eastern part of Ethiopia.

## Methods and Materials

### Study Setting and Period

The study was conducted among health facilities found in the Eastern Hararghe Zone, Oromia regional states found in the eastern part of Ethiopia. The East Hararghe zone has four hospitals, and 39 health centers according to the East Hararghe health bureau report (28). The study was conducted from October 25 to November 15, 2020. In 1998, the Ethiopian government established and recognized a health financing strategy that guides resources for the health sector to be organized from different sources and permits government to provide health services through its health facilities by means of a cost-sharing arrangement with users, providing services to the poor free of charge through a the fee-waiver system, as well as free provision of selected public health services (29). The Ethiopia COVID-19 public health emergency operation center (PHEOC) serves as a center for better coordinating the preparation, response, and recovery for public health emergencies. The center has been at the forefront of implementing efforts and mobilizing critical resources and lead house-to-house screenings and scaling up diagnostic testing. The center is collaboratively working with stakeholders: government agencies, partner organizations, UN agencies, embassies, hospitals, Industrial parks, and others (30).

### Study Design and Source Population

Health the facilities-based cross-sectional study design was used. All health care workers include nurses, medical doctors, and allied health professionals from ten randomly selected health facilities found in the Eastern Hararghe zone were the study population. Health care workers who were on annual leave and for other activities away from health facilities during the period of data collection were excluded.

### Sample Size Determination

The sample size was calculated using the formula for estimation of a single population proportion (*n* = [(Zα/2)2 ^*^P(1-P)]/d2) with the assumptions of 95% confidence level, marginal error (*d*) of 0.05, and 50.4% the prevalence of depression among health care workers (19). The total number of the health care provider in the zone was 928 thus, after applying the finite population correction formula and adding 10% of the non-response rate, the final sample size obtained was 297.

### Sampling Procedure and Sampling Technique

Two hospitals and eight health centers were selected by simple random sampling method (lottery method) from four hospitals and 39 health centers. All health care found in the selected health facilities was included.

### Data Collection and Questionnaire

An interview questionnaire was developed to assess socio-demographic characteristics include; age sex, marital status, educational level, type of profession, types of health facility, and worker experiences. The 9-item Patient Health Questionnaire (PHQ-9) was used to assess depression symptoms. The questionnaire was validated and developed by the local language, Afaan Oromo and Amharic language in the Ethiopian context and it recommended for use (31, 32).

The data was collected by ten nurses. Four MSC health professionals were assigned to supervise the data collection process. Both the data collectors and supervisors were taken 2-day intensive training before the actual work about the aim of the study, procedures, data collection techniques, the art of interviewing, ways of collecting the data, and clarification. Data were collected by interview technique, since the health care providers may have limited knowledge of psychiatric symptoms. The intensive supervision was done by the principal investigator, co-investigators, and supervisors. Completeness, accuracy, and consistency of data were checked at the site of data collection throughout the data collection period. Finally, double data entry was done by two data clerks, and the consistency of the entered data was cross-checked by comparing the two separately entered data.

### Measurements

The PHQ-9 is the depression module, which scores each of the nine DSM-IV criteria as 0–3 for every nine symptoms of depression, “0” not at all, “1” several days “2” more than half the days “3” nearly every day. Scoring was done by counting the number of boxes checked in a column. Multiply that number by the value indicated above, then add the subtotal to produce a total score. PHQ-9 total score for the nine items ranges from 0 to 27. Scores of zero, 1–4, 5–9, 10–14, 15–19, and 20–27 represent cut-points for no depression symptoms, minimal, mild, moderate, moderately severe, and severe depression, respectively (33). For logistic regression analysis we have categorized based on the PHQ-9 recommendation cut off point for depression, > 10 and coded as “1” and cut-off point ≤ 10 coded as “0.”

The age of the mother was recorded based on maternal response later and was grouped as 19–29 and ≥ 30 with codes 1 and 2, respectively, for analysis. Work experience was grouped <5 and ≥5 years with codes 1 and 2, respectively for analysis. Perceived susceptibility refers to a person's subjective perception of the risk of acquiring an illness or disease. Perceived severity refers to the subjective assessment of the severity of a health problem and its potential consequences (34). This study data for perceived susceptible was collected by interview using a single question “How likely do you think it is that you will develop COVID-19 during your providing care?” liker scale of five were used and labeled as “Strong unlikely,” “unlikely,” “Neutral,” “likely,” and “Strongly likely” and coded with 1, 2, 3, 4, and 5 respectively. For logistic regression analysis, we categorize as strong unlikely, unlikely, and neutral as no perceived susceptible, no susceptible coded as “1” and susceptible coded as “2.” With a similar pattern. Data for perceived severity was collected by interview using question with a single question “Getting COVID-19 in the future worries me and It is important for me to prevent getting.” Likert scale of five was used and labeled as “Strong unlikely,” “unlikely,” “Neutral,” “likely,” and “Strongly likely” and coded with 1, 2, 3, 4, and 5, respectively. For logistic regression analysis, we categorize as strong unlikely, unlikely, and neutral as no perceived susceptible, no susceptible coded as “1” and susceptible coded as “2.”

### Data Analysis

Double data entry was made using the Epidata 3.1 software. Then after validation was done the data exported to the STATA statistical package version 14 for further analysis. Descriptive statistics were used to summarize the variable. Continuous variables like age and work experience were first transformed into categorical variables before analyzed. Initially, the crude odds ratio (COR) along with a 95% confidence interval was estimated to assess the association between each independent variable and the outcome variable. Multicollinearity was tested using the Variance Inflation Factor (VIF) test and the tolerance test. No multicollinearity problem was found. The Hosmer-Lemeshow goodness-of-fit tests were used to test for model fitness (35). The logistic regression model was used to assess the association between predictor variables and the outcome variable depression symptoms. Adjusted Odds Ratio (AOR) along with a 95% confidence interval was estimated to assess the strength of the association. Statistical significance was declared at a *p*-value ≤ of 0.05.

### Ethical Consideration

The study was approved by the Haramaya University, College of Health and Medical Sciences Institutional Health Research Ethics Review Committee (IHRERC) with a reference number of IHRERC/230/2020. The permission and agreement consent was obtained from East Hararghe Zone, participating District health bureau, and health care facilities office prior to the study. The study participant was informed about the purpose of the study, their right to refuse, and written and signed voluntary consent was obtained prior to data collection. To maintain confidentiality, interviews were conducted in a separate room and all information obtained in this study was handled with anonymity. Based on the interpretation and recommendation of PHQ-9 author HCWs who had depression symptoms were referred to the psychiatric unit for further diagnosis and management who had moderate, moderate-severe, and severe symptoms.

## Results

### Socio-Demographic Characteristics and COVID-19 Related Perception of the Respondents

Of 297 total sample size, 265 health care workers participated in this study with a response rate of 89.22%. The reason for non-participation 13 (0.59%) were due to annual leave and 19 (0.71%) were due to additional assignments away from health facilities. The mean (±SD) age of HCWs was 29.29 (±6.40) years. The mean (±SD) HCWs work experience was 6.25 (±4.98) years. Of these, 161 (60.75%) were working in the inpatient department, the majorities 118 (44.53%) were nurses in the profession, and more than two-thirds of 183 (69.06%) were married. After categorizing, 202 (76.23%) and 222 (83.77%) respondents were perceived as susceptible and perceived severe to COVID-19, respectively (Table 1).

**Table 1:** Socio-demographic characteristics and COVID-19 related perception of respondents in in Eastern Hararghe zone, Oromia region, Eastern Ethiopia, 2020.

**Characteristics**	**Category**	**Frequency**	**Percent**
Age	19–29	170	64.15
	≥30	95	35.85
Work experience	<5 years	136	51.32
	≥5 years	129	48.68
Type of facility	Health center	167	63.02
	Hospital	98	36.98
Ward/area	Inpatient department	161	60.75
	Outpatient department	104	39.25
Profession	Allies of clinical	59	22.26
	Nurse	188	70.94
	Physician	18	6.79
Level of education	Diploma	101	38.11
	Bsc	144	54.34
	MSc/MPH	2	0.75
	Medical Doctors	18	6.79
Sex	Male	149	56.23
	Female	116	43.77
Marital status	Unmarried	82	30.94
	Married	183	69.06
Perceived susceptibility	No	63	23.77
	Yes	202	76.23
Perceived severity	No	43	16.23
	Yes	222	83.77

### Participants Response to PHQ-9 Questions

More than half, 148 (55.85%) of respondents not at all had interest or pleasure in doing things over the past 2 weeks. Two third 174 (65.66%) of the respondents not at all feeling down, depressed, or hopeless, feeling tired 171 (64.53%) and trouble concentrating on things, such as reading the newspaper or watching television 173 (65.28%) over the past 2 weeks (Table 2).

**Table 2:** Response of the participant to each depression question by health care workers in Eastern Hararghe zone, Oromia region, Eastern Ethiopia, 2020.

**Characteristics**	**Not at all**		**Several days**		**More than half the days**		**Nearly every day**	
	***n***	**%**	***n***	**%**	***n***	**%**	***n***	**%**
Loss of interest	148	55.85	54	20.38	34	12.83	29	10.94
Feeling depressed	174	65.66	42	15.85	37	13.96	12	4.53
Sleep problems	196	73.96	27	10.19	31	11.70	11	4.15
Loss of energy	171	64.53	44	16.60	41	15.47	9	3.40
Appetite problems	208	78.49	23	8.68	23	8.68	11	4.15
Self-blame	214	80.75	22	8.30	20	7.55	9	3.40
Concentration problems	173	65.28	48	18.11	22	8.30	22	8.30
Agitation/retardation	193	72.83	38	14.34	24	9.06	10	3.77
Suicidal ideations	234	88.30	16	6.06	10	3.77	5	1.89

### Level of Depression of Participants

**Figure 1:**
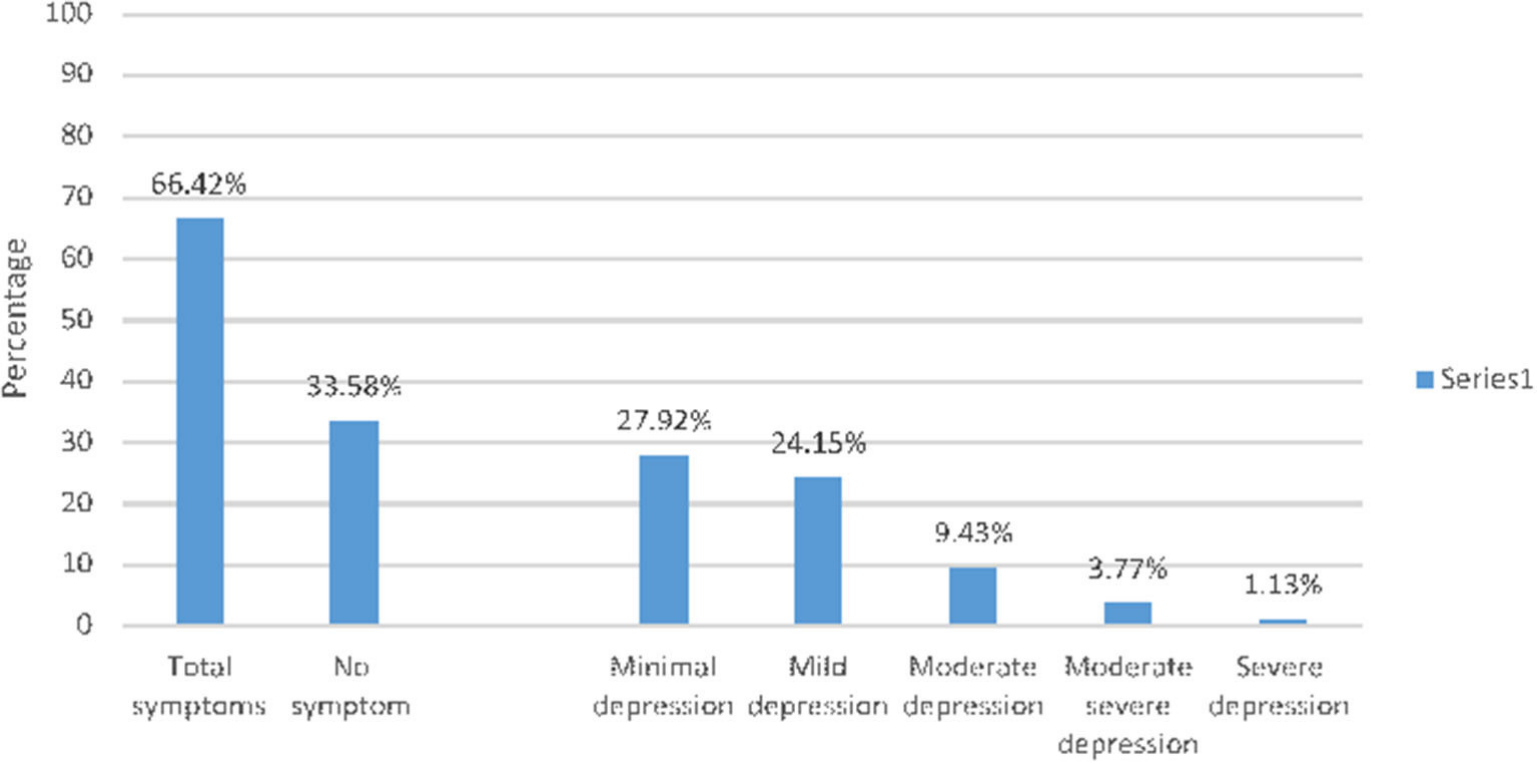
Level of depression among health care workers in Eastern Hararghe zone, Oromia region, Eastern Ethiopia, 2020.

According to the cut-off defined by PHQ-9 the level of depression of respondents were calculated. Of the total 265 interviewed, 176 (66.4%, 95%CI: 60.4%-71.8%) of them reported at least one depressive symptom. Of respondents who had symptoms of depression, 74 (27.92%) had minimal, 64 (24.15%) had mild depression, 9.43% moderate depression, 3.77% had moderate severe depression, and 1.13% had severe depression (Figure 1).

### Predictors of Depression

The multivariable logistic regression analysis revealed the odds of depression was 2.34 times higher among female participants compared to male participants (AOR: 2.34, 95%CI: 1.09-5.02). In addition, the odds of depression for participants who perceived susceptibility to COVID-19 was 4.05 times higher among their counterpart (AOR: 4.05, 95%CI: 1.12-14.53) (Table 3).

**Table 3:** Factors associated with the depression of health care workers in Eastern Hararghe zone, Oromia region, Eastern Ethiopia, 2020.

		**Depression**			
**Characteristics**	**Category**	**No *n* (%)**	**Yes *n* (%)**	**COR (95 % CI)**	**AOR (95 % CI)**
Marital status	Unmarried	67 (81.75)	15 (18.29)	1	1
	Married	160 (87.43)	23 (12.57)	0.64 (0.31, 1.30)	0.59 (0.26, 1.32)
Age	19–24	144 (84.71)	26 (15.29)	1	1
	25–29	83 (87.37)	12 (12.63)	0.80 (0.38, 1.67)	0.89 (0.37, 2.13)
Sex	Male	133 (89.26)	16 (10.74)	1	1
	Female	94 (81.03)	22 (18.97)	1.94 (0.96, 3.9)^*^	2.34 (1.09, 5.02)^*^
Level of education	Diploma	137 (84.57)	25 (15.43)	1	1
	BSc and above	90 (87.38)	13 (12.62)	0.79 (0.38, 1.62)	0.65 (0.28, 1.43)
Type of facility	Health center	142 (85.54)	24 (14.46)	1	1
	Hospital	85 (85.86)	14 (14.14)	0.97 (0.47, 1.98)	0.80 (0.35, 1.83)
Type of profession	Allied profession	50 (84.75)	9 (15.25)	1	1
	Nurse	162 (86.17)	26 (13.83)	0.89 (0.39, 2.02)	0.96 (0.39, 2.37)
	Physicians	15 (83.33)	3 (16.67)	1.11 (0.26, 4.6)	1.06 (0.21, 5.18)
Work Experience		116 (85.29)	20 (14.71)	1	1
		111 (86.05)	18 (13.95)	0.94 (0.47, 1.87)	1.15 (0.50, 2.65)
Susceptibility	No	60 (60)	3 (4.76)	1	1
	Yes	167 (82.67)	35 (17.33)	4.19 (1.2, 14.13)	4.05 (1.12, 14.53)^*^
Severity	No	40 (93.02)	3 (6.98)	1	1
	Yes	187 (84.23)	35 (15.77)	2.49 (0.73, 8.51)	1.34 (0.35, 5.10)

*COR, crudes odds ratio; AOR, adjusted odds ratio; CI, Confidence interval;*

* *showed association*.

## Discussion

In this study, overall, 176 (66.42%) of the study participants have had at least one depression symptoms. Factors significantly associated with depression symptoms were being a female and perceived susceptibility to COVID-19.

The depression symptoms reported in this study were high. This may due to, Ethiopia is one of the low-income countries which left HCWs with high fear of COVID-19 due to lack of personal protective equipment (PPE) and shortage of human power to handle ever-increasing the case (14, 36). This may result in fears and concerns for self, family members, problems with children and spouses, and difficulties entailed in working at home are some of the stressors during the Covid-19 pandemic (37). As some bodies experience stress, adrenal glands make and release cortisol hormone into the bloodstream. Prolonged elevated cortisol level leads to the manifestation of depression (38–40) and minimize the body immunity (41). Health care providers at higher risk of experiencing severe disease and even may fatal. ICN confirms 1,500 nurses have died from COVID-19 in 44 countries and estimates that healthcare worker COVID-19 fatalities worldwide could be more than 20,000 as of October 28, 2020 (42). Without addressing mental health services, we risk not having a workforce left to care for us.

The finding was consistent with those reported in a study conducted in Poland 70.7% (43). However, it was higher than studies finding in china at 54.65% (4) and 44.37% (44). This is may be due to Ethiopia is one of the developing countries which left HCWs with high fear of COVID-19 due to lack of personal protective equipment (PPE) and shortage of human power to handle ever increasing the case (14, 36) and low preparedness to compact the pandemic (36, 45, 46). In contrast, it was lower than a study finding in Egypt were 77.2% (23) participants had symptoms of depression. This difference might be due to contextual differences.

Regarding symptoms of depression, majority 24.15% participants had mild a symptom of depression followed by moderate 9.43%, severe 3.77% and 3 extremely severe. This was almost in line with study finding in the United Kingdom where 32.1% participants had mild, 17% moderate, 8.9% moderate severe and 5.7% (47) had severe symptoms of depression, respectively. But, it was relatively lower than study finding in Pakistan, 18.18% mild, 20.91% moderate, 40.91% severe and 11.81% participants had extremely severe symptoms of depression (16). Also, it was lower than study finding in India, 7.3% had severe symptoms of depression (48). This difference may be due to high fear for contacting the infection as a result of insufficient PPE, and low preparedness to compact the pandemic (36, 45, 46).

The study showed depression were associated with female health care workers. This is supported by studies conducted in China (19, 49), Iran (24), Egypt (23), and Ethiopia (50). Women are among the highly vulnerable population groups during a pandemic due to disrupt the food system and other economic activities. A study in India showed COVID-19 case fatality rate among men were 2.9 and 3.3% among women (51). Study showed that, there was a gender difference in stress and coping styles, numerous studies have determined that women find themselves in stressful circumstances more often than men, and their coping style is more emotion-focused than that of men (52). Social roles also seem relevant in the stressful life experiences of women, especially in low-income countries. There can be sex differences in the use of coping strategies which is important for the mitigation of depressive symptoms (53). The study shows that certain psychological and social determinants were associated with increased depressive symptoms, women are disadvantageous in a low-income country, COVID-19 enhances the vulnerability to the mental health challenge (54). Measures must be put into place to ensure the protection of women on the frontlines while reducing COVID-19 deaths and adverse health effects among displaced populations.

Perceived susceptibility (perception of the risk of acquiring COVID-19) was associated with participants who had symptoms of depression. This is consistent with the study finding in China (22, 55). Perceived susceptibility may be related to shortages of personal protective equipment (PPE) or other essential equipment, low community role in practice to protect themselves and the play their role in mitigating the transmission including to the health care worker (56). Contradicting of belief in susceptibility, with the low accessibility of protective measure, less concern of the community and the health manager may put health care providers at risk of experiencing depressive symptoms. Health care may also face moral dilemmas in decision-making around the provision of care with limited resources (6).

Depression symptoms were related to work absences and reduced work performance. The study investigated that workers with depression may benefit from care involving medical and psychological interventions (26). World Health Organization identified protecting the mental well-being of healthcare workers caring for people with COVID-19 has been recognized as crucial for the long-term capacity of the health workforce (27). However, most health care workers experiencing depression were not planning to seek help and not aware of workplace help plan (26). Prioritizing the well-being of health care workers must be at the forefront of the action plan to tackle the coming wave of this pandemic. We suggested that comprehensive psychological assistance should be provided to support the mental well-being of healthcare workers.

The trend of the number of COVID-19 confirmed cases in Ethiopia revealed that another wave of the pandemic is happening. The current increment in the number of COVID-19 cases may be attributed to the spread of the disease in the community due to relaxation of public health and social measures (PHSM) and fatigue around adhering to PSHM measures compounded by a high risk of importation variants of concern (VOC) (30). Urgent and effective community based intervention may reduce the pressure on mental health of the health care workers.

## Strengths, Limitations, and Future Directions

The study conducted in multiple health care facilities including Hospitals and Health Centers found in town and rural areas. We never used design effect to increase the power due to budget constraints. However, the cross-sectional the study is important to be aware of the predictive, there is generally no evidence of a temporal relationship between exposure and outcome because the exposure and outcome are simultaneously assessed. The study includes only depressive symptoms, other mental health problems were not included. This study also not include staff who had contact with COVID-19 patients. We suggest a study that includes health professionals who had contact with COVID-19 patients, assessment of other mental health problems, longitudinal studies that assess the prolonged implication on HCWs need further investigation and more sample size. Finally, unaccounted and residual confounding could have had an effect on the association found.

## Conclusion

The magnitude of health care workers experienced depression was high. Health care workers' mental health needs to be protected during the COVID-19 pandemic. Female health care workers, and health care workers perceived susceptibility of COVID-19 need attention.

## Data Availability Statement

The raw data supporting the conclusions of this article will be made available by the authors, if officially requested without reservation.

## Ethics Statement

The study was reviewed and approved by the Institutional Health Research Ethics Review Committee of the Colleges of Health and Medical Sciences, Haramaya University. Written consent was obtained from each participants before commencement of data collection. To maintain confidentiality, interviews were conducted in a separate room and all information obtained in this study was handled with anonymity.

## Author Contributions

YD and BB conceived and designed the paper, involved in data collection, performed the statistical analysis, interpret the results, and wrote and reviewed the manuscript. All authors contributed to the article and approved the submitted version.

## Conflict of Interest

The authors declare that the research was conducted in the absence of any commercial or financial relationships that could be construed as a potential conflict of interest.
